# Zika Virus: Recent Advances towards the Development of Vaccines and Therapeutics

**DOI:** 10.3390/v9060143

**Published:** 2017-06-13

**Authors:** Monica A. McArthur

**Affiliations:** Center for Vaccine Development, University of Maryland School of Medicine, Baltimore, MD 21201, USA; mmcarthu@som.umaryland.edu; Tel.: +1-410-706-5328

**Keywords:** Zika virus, animal models, immunology, vaccines, congenital Zika syndrome

## Abstract

Zika is a rapidly emerging public health threat. Although clinical infection is frequently mild, significant neurological manifestations have been demonstrated in infants born to Zika virus (ZIKV) infected mothers. Due to the substantial ramifications of intrauterine infection, effective counter-measures are urgently needed. In order to develop effective anti-ZIKV vaccines and therapeutics, improved animal models and a better understanding of immunological correlates of protection against ZIKV are required. This review will summarize what is currently known about ZIKV, the clinical manifestations and epidemiology of Zika as well as, the development of animal models to study ZIKV infection, host immune responses against ZIKV, and the current state of development of vaccines and therapeutics against ZIKV.

## 1. Introduction

As of March 2017, 84 countries and territories have reported evidence of mosquito-borne Zika virus (ZIKV) transmission, including the United States of America [1]. Furthermore, many of these countries have reported microcephaly and other central nervous system (CNS) malformations potentially associated with ZIKV infection, or suggestive of congenital infection as well as increased incidence of Guillain–Barré syndrome (GBS) and/or laboratory confirmation of ZIKV infection among GBS cases [1]. The temporal association of ZIKV transmission with clusters of microcephaly and GBS was declared a Public Health Emergency of International Concern (PHEIC) by the World Health Organization (WHO) on 1 February 2016, a designation which was subsequently ended at the fifth meeting of the Emergency Committee on ZIKV, microcephaly and other neurological disorders on 18 November 2016 [2,3]. According to the WHO, the decision was made to escalate the coordination and response to ZIKV to a more sustained program to address the long-term nature of the disease and its consequences [2]. As ZIKV continues to spread, many “unknowns” remain and considerable research is needed to advance our understanding of this important pathogen. The current literature pertaining to the clinical manifestations and epidemiology of ZIKV as well as, the development of animal models to study ZIKV infection, host immune responses against ZIKV, and the current state of development of vaccines and therapeutics against ZIKV will be discussed.

## 2. Isolation and Characterization

ZIKV was first isolated from a sentinel rhesus monkey in Uganda, near the Zika forest, in 1947 [4]; however, there was limited documentation of human infection prior to the 2007 outbreak on Yap Island in the Federated States of Micronesia [5]. ZIKV belongs to the family *Flaviviridae*, genus *Flavivirus* and is a mosquito-borne virus in the Spondweni group [6]. Flaviviruses are among the most medically significant arboviruses and include, in addition to ZIKV, pathogens such as yellow fever virus (YFV), Japanese encephalitis virus (JEV), West Nile Virus (WNV), tick-borne encephalitis virus (TBEV), and the four dengue viruses (DENV1–4).

Flaviviruses are enveloped RNA viruses that contain the viral genome complexed with multiple copies of the capsid protein (C) and surrounded by an icosahedral shell composed of 180 copies of the envelope (E) glycoprotein and the membrane (M) or precursor membrane (prM) proteins. The E and M/prM proteins are anchored in a lipid membrane [7]. The full-length genome of ZIKV is 10,794 nucleotides and encodes 3419 amino acids, which, in addition to the structural proteins (C, prM, and E), constitute seven non-structural proteins (NS1, NS2a, NS2b, NS3, NS4a, NS4b, and NS5) [6,7]. The non-structural proteins are involved in replication and assembly of the virus as well as antagonizing the host innate immune response [7]. Specifically, NS3 and NS5 are large, multifunctional proteins which contain several enzymatic activities involved in polyprotein processing (NS3) and RNA replication (NS3 and NS5) [8]. Additionally, NS5 has been demonstrated to antagonize the interferon (IFN) response [9].The crystal structure of ZIKV has been solved and demonstrates that the mature ZIKV structure is similar to mature WNV and DENV structures [7]. However, there are notable differences in the E protein structure which may be responsible for cellular tropism and contribute to disease outcomes [7]. 

Phylogenetic analyses of ZIKV strains isolated from 1947 to 2016 identified two distinct clades (lineages), African and Asian [10]. The causative agent of the current ZIKV epidemic belongs to the Asian lineage, which, while not closely related to the African lineage, shares a common ancestor [10]. Comparisons of the E protein sequences from the two lineages found that the Asian lineage contains insertions in the E protein glycosylation motif which are not present in the African lineage [10]. When the amino acid sequences of the E protein of ZIKV isolates from human (2015/2016 epidemic), monkey (pre-epidemic), and mosquito (pre-epidemic) were compared, a total of sixteen amino acid substitutions were identified, resulting in subtle structural changes [10]. These changes, although subtle, may impact ZIKV virulence and host tropism [10].

## 3. Epidemiology

Despite relatively limited reports of human cases of ZIKV infection from 1947 until the 2007 outbreak on Yap Island, ZIKV has now spread dramatically to include over 80 countries and territories with vector-borne transmission [1]. Following the introduction of ZIKV to Yap Island, sporadic cases of ZIKV infection continued to be reported in Southeast Asia during the mid-2010s [11]. A major epidemic of ZIKV infection ensued in French Polynesia in 2013–2014 and some severe cases were associated with neurological complications such as GBS [12,13]. ZIKV was first reported in Brazil in 2015 with large numbers of suspected cases and the initial observation that the number of newborn infants with microcephaly was increased in ZIKV-affected areas later that year [11]. ZIKV has since spread to include much of Central and South America and the Caribbean. Furthermore, vector-borne transmission of ZIKV has been documented in the US in both Texas and Florida [14]. 

The primary mode of ZIKV transmission is through the bite of infected mosquitoes, with *Aedes aegypti* and *Aedes albopictus* being the predominant vectors [6,15]. While *Ae. aegypti* mosquitoes are confined to tropical and sub-tropical regions, *Ae. albopictus*, are distributed throughout tropical, sub-tropical, and temperate regions [16]. Since 2015, ZIKV has spread rapidly through the range occupied by *Aedes* mosquitos in the Americas [15]. In addition to vector-borne transmission, sexual transmission is a contributor to ZIKV spread [17,18,19,20]. Additional modes of transmission have also been reported, including transmission from mother to child, blood transfusion-related transmission, laboratory transmission, and transmission by physical contact [16]. 

It is likely that ZIKV will continue to spread; however, it is unclear at this time whether ZIKV transmission will remain epidemic, eventually becoming episodic with intervening periods of relative inactivity, or if it will become endemic with seasonal transmission patterns [21]. Characterization of the epidemiology of ZIKV is complicated by inadequate diagnostic assays (discussed below) and co-circulation with viruses demonstrating similar clinical presentations (i.e., Chikungunya virus (CHIKV) and DENV) [22].

## 4. Clinical Manifestations

Most individuals, ~80%, infected with ZIKV are asymptomatic [23]. When symptoms are present, they are generally mild and may include pruritic maculopapular rash, fever (typically low-grade), arthritis, arthralgia, non-purulent conjunctivitis, and edema of the extremities [5,23,24,25]. Additional symptoms of headache, myalgia, retro-orbital pain, low-back pain, lymphadenopathy, and vomiting have also been reported [5,24]. While disease is frequently mild, fatal cases have been reported, particularly in individuals with underlying medical conditions [26,27,28,29,30,31]. 

### 4.1. Neurological Manifestations of ZIKV Infection

A major concern during the current ZIKV epidemic has been the association of ZIKV with neurological complications. GBS is the most frequent neurological sequela reported [12,13,25,32]. Although GBS is commonly thought to have a good prognosis, up to 20% of GBS patients may remain severely disabled and even with treatment, approximately 5% of GBS cases are fatal [33]. GBS is characterized by progressive bilateral and relatively symmetric weakness of the limbs frequently with hyporeflexia or areflexia. There is considerable evidence suggesting GBS has an autoimmune etiology [33]. GBS has previously been associated with preceding infections such as *Campylobacter jejuni*, cytomegalovirus (CMV), Epstein–Barr virus (EBV), varicella zoster virus (VZV) and *Mycoplasma pneumoniae*. Additionally, there has been a link to influenza vaccination [34]. The mechanism by which ZIKV infection is associated with GBS remains unknown. It is therefore of critical importance to monitor for GBS in large scale vaccine trials as well as natural infection.

Other neurological complications associated with ZIKV infection including encephalitis, meningoencephalitis, and acute myelitis have also been reported [30,35,36].

### 4.2. Congenital Zika Syndrome

The Brazil Ministry of Health reported an unusual increase in cases of microcephaly in October and November 2015, the Pan American Health Organization (PAHO) and WHO requested Member States to monitor for similar events [37]. The identification of an increased number of cases of microcephaly potentially associated with ZIKV prompted the declaration of a PHEIC in February 2016 [3]. Subsequently, multiple reports of microcephaly and other birth defects following ZIKV infection during pregnancy have been made [38,39,40,41,42,43]. Following recognition of increases in the number of infants with microcephaly in Brazil [44] and French Polynesia [40] which were spatially and temporally associated with outbreaks of ZIKV infection, a causal link was suspected [45]. Subsequently, Rassmussen et al. applied Shepard’s criteria for causality and suggested that sufficient evidence had been accumulated to infer a causal relationship between ZIKV infection and microcephaly as well as other brain anomalies [46]. Evidence to support the role of ZIKV infection during pregnancy in causing microcephaly and other neurological malformations includes: (a) temporal association (with a six-month delay) of increases in microcephaly with increased ZIKV transmission [44]; (b) data modeling indicating the first trimester is the primary risk period of pregnancy [40]; (c) identification of ZIKV in the brains of fetuses and infants (who died) with microcephaly [47,48]; (d) attenuation of human neural progenitor cell growth following in vitro ZIKV infection [49]; (e) in vitro infection of placental macrophages and cytotrophoblasts [50]; and (f) evidence of microcephaly associated with ZIKV infection in mouse and non-human primate models [51,52,53,54]. 

## 5. Animal Models

Animal models play an important role in understanding viral pathogenesis, identifying promising vaccine candidates, and testing therapeutics. The scientific community has exerted considerable effort in developing animal models which recapitulate various aspects of human ZIKV infection and disease. For a comprehensive review of animal models for ZIKV infection and Zika disease, see Morrison and Diamond [55].

### 5.1. Rodent Models

Initial attempts to infect wild-type (wt) mice with ZIKV resulted in infection only by the intra-cerebral (ic) route [4]; however, passage through mouse brain resulted in adaptation to the mouse model [56]. Similarly, guinea pigs and rabbits did not show signs of disease following inoculation with ZIKV [56]. Although adult immunocompetent mice do not typically develop overt signs of clinical disease, wt mice have been used to demonstrate intrauterine infection [52,57]. Additionally, treatment of wt mice with antibodies blocking aspects of the interferon (IFN) response have been used as described below [54,58,59]. More recently immunocompromised mouse models, mostly lacking IFN responses, have been utilized to study systemic infection with ZIKV [54,59,60,61,62]. A variety of mouse models which lack various components of the IFN response have been employed, including A129 (lack IFN α/β receptor and are, therefore, incapable of responding to Type I IFN) [61,62], AG129 (lack Type I and II IFN responsiveness) [60,62], *Irf3*^−/−^ *Irf5*^−/−^ *Irf7*^−/−^ (triple knockouts which produce very little IFN α/β) [59], *Ifnar1*^−/−^ (lack IFN-α/β receptor) [54,59]. Interestingly, while Lazear et al., reported severe neurological disease in *Irf3*^−/−^ *Irf5*^−/−^ *Irf7*^−/−^ triple knock-out (TKO) and *Ifnar1*^−/−^ mice following subcutaneous inoculation with either a contemporary human isolate from French Polynesia (H/PF/2013) or the original Ugandan ZIKV strain (MR766), they found no overt disease in *Irf3*^−/−^, *Irf* ^−/−^, and *Mavs*^−/−^ single knock-out mice [59]. Despite the fact that both H/PF/2013 and MR766 elicited neurological disease in these models, H/PF/2013 demonstrated greater pathogenicity than the original Ugandan strain, MR766. Following intravenous inoculation, *Irf3*^−/−^ *Irf5*^−/−^ *Irf7*^−/−^ TKO were more susceptible to ZIKV infection than *Ifnar1*^−/−^ mice indicating a potential role for IRF-3-dependent, IFN-α/β-independent restriction mechanisms [59]. Furthermore, wt mice treated with MAR1-5A3 (a monoclonal antibody (mAb) that blocks the IFN-α/β receptor (IFNAR)) were susceptible to ZIKV infection but did not demonstrate a same neurological phenotype as *Ifnar1*^−/−^ mice [59]. Additionally, mice immunosuppressed by treatment with dexamethasone developed ZIKV infection when inoculated with a clinical ZIKV isolate from Puerto Rico (PRVABC59) and treatment with type I IFN resulted in improved clinical outcome [63]. Taken together, these studies indicate an important role for the IFN response in protection against ZIKV infection.

Perhaps even more important than models of systemic ZIKV infection, are models of ZIKV infection during pregnancy. Multiple mouse models for ZIKV infection during pregnancy have been proposed. The *Ifnar1*^−/−^ and MAR1-5A3 blocking models were used by Miner et al. to investigate ZIKV replication and trans-placental transmission in pregnant dams following infection with a clinical isolate from French Polynesia (H/PF/2013) [54]. Infection of *Ifnar1*^−/−^ dams mated with wt males resulted in fetal demise, while when pregnant wt dams were given anti-IFNAR mAb prior to and during infection, there was mild intrauterine growth restriction (IUGR) and viral infection within the fetal head during a key period of neurodevelopment [54]. Another study using wt mice (SJL and C57BL/6 strains) found that infection of pregnant SJL mice with a Brazilian strain of ZIKV intravenously (iv) resulted in IUGR and ZIKV RNA was identified in multiple fetal tissues, particularly the brain [52]. Furthermore, surviving fetuses demonstrated cortical malformations with reduced cell number and cortical layer thickness (both signs associated with microcephaly in humans) as well as ocular abnormalities [52]. Adult C57 mice inoculated intra-peritoneally (ip) with a contemporary clinical ZIKV isolate were also able to transmit virus to their unborn fetuses and resulted in infection of primary neural progenitor cells responsible for cortex development as well as reduction of these cortex founder cells in the fetuses [57]. Intravaginal exposure to a Cambodian ZIKV strain, FSS13025, in both wt and *Ifnar1*^−/−^ mice demonstrated replication of the virus in the vaginal mucosa (higher levels were identified in *Ifnar1*^−/−^ mice) which resulted in IUGR and fetal brain infection (wt mice) and severe IUGR and fetal death (*Ifnar1*^−/−^ mice) [64]. Direct infection of embryonic brains has also been demonstrated, and an Asian ZIKV strain, SZ01, was found to infect neural progenitor cells and cause cell death resulting in microcephaly [53]. Additionally, transcriptomic analyses following ZIKV infection demonstrated enrichment of up-regulated immune-response-related and apoptosis pathways providing further evidence that cytokines may play a role in ZIKV pathogenesis [53]. In sum, studies in mice have demonstrated that the interferon response is likely to play a critical role in protection from or susceptibility to ZIKV infection. While these studies support a causal role for ZIKV infection during pregnancy and microcephaly/neurological malformations, it is important to note that there are considerable differences in the morphological, spatial, and temporal placentation between mice and humans as well as differences in in utero brain development [65,66]. Further efforts to develop novel and enhance existing animal models to better recapitulate human disease are needed.

### 5.2. Non-Human Primate (NHP)Models

While mice represent a relatively easy to study small animal model for ZIKV, the previously mentioned differences between humans and mice necessitate additional models of disease. Immunocompetent macaque monkeys have similar gestation and fetal development to humans providing an animal model that more faithfully recapitulates human disease [51,67,68,69]. In non-pregnant rhesus macaques, the duration of detectable viral RNA in plasma is similar to that in humans, 6–7 days post infection, and the decline in plasma levels of viral RNA is coincident with increases in anti-ZIKV neutralizing antibody titers [69]. Dudley et al., have recently demonstrated prolonged viremia following infection with an Asian lineage ZIKV in pregnant rhesus macaques compared to non-pregnant animals [67]. Furthermore, they identified ZIKV-specific proliferation of natural killer (NK) cells and adaptive immune responses, including proliferation of CD4+ and CD8+ T cells, circulating plasmablasts, and ZIKV-specific IFN-γ production (by Elispot) [67]. ZIKV RNA has also been detected in placenta, fetal brain and liver as well as maternal tissues following infection of a pigtail macaque with an Asian lineage ZIKV [51]. Additionally, Magnetic Resonance Imaging (MRI) abnormalities in the fetal brain were identified 10 days after inoculation suggesting that fetal brain injury begins shortly after infection [51]. In a recent study by Osuna et al., both rhesus and cynomolgus macaques were shown to be highly susceptible to ZIKV infection following subcutaneous (sc) inoculation with isolates of Thai and Puerto Rican origin [68]. Activation of T and B cells was reported and multiple animals developed T cells specific for ZIKV peptides as evidenced by cytokine production following in vitro stimulation [68]. Studies utilizing non-human primate models to test anti-ZIKV vaccine candidates will be discussed below.

## 6. Immunology

### 6.1. Innate Immunity

Early responses by the innate immune system are the first line of host defense for the suppression of viral infections. IFN production is a major component of the innate response and the transcriptional regulation of numerous IFN-regulated genes leads to an antiviral environment [70]. As previously discussed, mice deficient in various components of the IFN response have increased susceptibility to ZIKV infection compared to wt mice [54,59,60,61,62,64]. It has been shown that human primary trophoblast cells, isolated from full-term placentas, constitutively release antiviral type III IFNλ1 which acts in both autocrine and paracrine fashion to protect cells from ZIKV infection [71]. These in vitro data suggest that ZIKV may not access the fetal compartment by direct replication in placental syncytiotrophoblasts during later stages of pregnancy [71]. However, it remains to be determined whether first-trimester trophoblasts are more permissive to ZIKV infection than late pregnancy villous trophoblasts and/or the antiviral effects of IFNλs [71]. Importantly, there are significant differences in the type III IFN pathway between mice and humans potentially complicating the interpretation of mouse models of ZIKV during pregnancy [71]. Flaviviruses have previously been shown to antagonize IFN signaling through multiple mechanisms [72]. In vitro studies have demonstrated that ZIKV NS5 binds to and targets the human IFN-regulated transcriptional activator STAT2 for proteasomal degradation; in contrast, mouse STAT2 is refractory to ZIKV NS5 [73]. NS5-mediated degradation of STAT2 has also been demonstrated for DENV, but the mechanisms of degradation differ between DENV and ZIKV [73]. Interestingly, although Type I and Type III IFN use different cell-surface receptors they both signal through the Janus kinases-signal transducers and activators of transcription (Jak-STAT) pathway, including STAT2 [73,74]. For this reason, it is possible that while, Type III IFN are produced by villous trophoblasts [71], ZIKV may be able to evade Type III IFN signaling by degrading STAT2 mediated by NS5 [73]. In a mouse model of intravaginal ZIKV infection, it was demonstrated that antiviral Type I and III IFN and other inflammatory mediators were poorly induced and that there was robust viral replication in the vaginal mucosa [75]. Viral replication has also been shown in the vaginal mucosa of wt pregnant mice with even higher levels of viral replication demonstrated in *Ifnar1*^−/−^ mice [64]. Furthermore, Khan et al. found that if the dampened innate immune response was augmented (either by systemic infection with an unrelated pathogen or vaginal administration of acitretin) ZIKV replication was inhibited [75]. Taken together with studies using various strains of IFN-deficient mice, these data indicate a critical role for IFN in resistance to ZIKV infection.

### 6.2. Humoral Immunity

Neutralizing antibodies are thought to be a major factor in the protection against ZIKV infection [76,77,78]. In fact, broadly neutralizing human monoclonal antibodies directed against the E protein were protective against ZIKV-infection in type I/II interferon receptor-knockout mice [78]. However, these mAb were initially derived from DENV-infected individuals, not ZIKV-infected individuals. In another study, Sapparapu et al. isolated a panel of human mAb from individuals infected with ZIKV and identified a potent neutralizing antibody recognizing a quaternary epitope of the E protein dimer-dimer interface [76]. This mAb was also found to protect anti-IFNAR1 treated C57BL/6 mice from challenge with a mouse-adapted strain of ZIKV [76]. Furthermore, passive transfer of anti-ZIKV antibodies elicited by immunization has been shown to be protective in mice [79,80]. 

Antibodies against flaviviruses tend to be highly cross-reactive [81] and ZIKV is no exception. In fact, serological diagnosis has been complicated by a lack of ZIKV-specific antibody based assays. Both ELISA and Plaque Reduction Neutralization Tests (PRNT) demonstrate significant cross-reactivity with related viruses, including DENV which co-circulates in many of the areas affected by the current ZIKV epidemic [82]. Multiple groups have identified antibodies from DENV patients, which cross-react, and in some cases, neutralize ZIKV [78,83,84,85,86]. Barba-Spaeth et al. identified a subset of ZIKV-neutralizing antibodies that target a conformational epitope and reported the crystal structure of two of these antibodies in complex with the ZIKV E protein to reveal an epitope conserved between DENV and ZIKV [83]. These structural details of a conserved quaternary epitope may provide an important antigenic target for the development of cross-protective vaccines [83].

Although neutralizing antibodies may provide protection against ZIKV infection, there is also concern for antibody dependent enhancement (ADE) which may occur due to cross-reactive, poorly neutralizing antibodies. ADE is an immunological phenomenon in which non-neutralizing or weakly neutralizing antibodies facilitate viral entrance into Fc-receptor bearing cells such as monocytes and macrophages and has been described extensively in the context of secondary DENV infection [87,88,89,90]. While there has, to date, been no indication in epidemiological studies that prior DENV infection results in ADE of ZIKV infection or disease, in vitro analyses of ZIKV cross-reactive antibodies from DENV-infected individuals have demonstrated enhanced ZIKV infection through an Fc receptor-mediated process [84,86]. In AG129 (immunocompromised) mice, a DENV cross-reactive anti-ZIKV mAb enhanced DENV2 infection and disease; however, the LALA version of the mAb, which lacks the ability to bind to the Fc receptor, did not enhance disease [77]. While evidence of ADE has been demonstrated in vitro and in mouse models, extensive epidemiological studies are required to determine the clinical significance of ADE in areas where ZIKV and DENV co-circulate.

### 6.3. Cell Mediated Immunity

While significant information is emerging regarding humoral responses against ZIKV, there have been relatively few studies that explore cell mediated immune (CMI) responses against ZIKV. Data from DENV infection have demonstrated that T cell responses contribute to protection and/or disease enhancement [91,92,93,94,95]. Similarly, strong CD4+ and CD8+ T cell responses against the live-attenuated YFV vaccine, 17D have been established [96,97,98]. CMI responses against flaviviruses including JEV and WNV have also been reported [99,100]. Rivino et al. presented the argument that due to the high degree of sequence homology among DENV and ZIKV, some of the HLA-restricted CD8+ T cell epitopes may be conserved [92].

ZIKV-specific T cell responses have been elaborated in animal models [67,68,80,101,102,103] and humans [77]. In the NHP model, both CD4+ and CD8+ T cells were expanded as plasma viral RNA loads decreased following infection of rhesus macaques with an Asian lineage ZIKV strain. Additionally, IFN-γ secretion was detected in response to in vitro stimulation of peripheral blood mononuclear cells (PBMC) isolated from infected animals with overlapping peptides from the ZIKV NS5 [67]. Furthermore, increased expression of CD69 (a marker of early T cell activation) by T cells occurred following ZIKV infection and peaked between Days 2 and 5 post-infection [68]. ZIKV-specific cytokine production by CD4+ and CD8+ T cells was identified in both PBMC and lymph nodes of ZIKV-infected NHP at multiple time-points post-infection with peak responses noted on day 28 post-infection [68]. Elong Ngono et al. recently used mouse models to begin to dissect the CD8+ T cell responses against ZIKV[58]. They identified increases in antigen-experienced CD8+ T cells in mice infected with both African (MR766) and Asian (FSS13025) strains of ZIKV [58]. Moreover, they identified ZIKV-derived epitopes recognized by CD8+ T cells which encompassed peptides from the majority of the ZIKV proteins with a predominance of E protein-derived epitopes [58]. These ZIKV-responsive CD8+ T cells were multifunctional and also found to mediate cytotoxicity [58]. Furthermore, depletion of CD8+ T cells resulted in increased levels of infectious ZIKV in the serum and multiple tissues, including the brain [58]. In humans, CD4+ T cell responses against the ZIKV NS1 protein have been demonstrated [77]. These T cell responses were primarily in the CXCR3+ compartment and were poorly cross-reactive with DENV [77]. Although much remains to be learned about the T cell responses against ZIKV, current data support an important role for T cells in protection against ZIKV. 

## 7. Diagnostics

The development of diagnostic tests for ZIKV has proven challenging due, in part, to serological cross-reactivity among flaviviruses [104,105]. There are currently no US Food and Drug Administration (FDA) approved ZIKV diagnostic tests; however, both serologic and nucleic acid amplification tests (NAAT) including the immunoglobulin (Ig)M class capture enzyme-linked immunosorbent assay (MAC-ELISA) and the Trioplex reverse transcription polymerase chain reaction (RT-PCR) have been given Emergency Use Authorization (EUA) by the FDA [106]. In addition to these diagnostic tests given EUA, there are numerous research-based assays; however, these assays do not provide validation using the most recent viral strains and fully documented clinical specimens [104]. 

### 7.1. Serological Tests

Due to the complexities of serological diagnostic testing for ZIKV infection, interim guidance for the interpretation of ZIKV antibody test results has been published [107]. Huzly et al. demonstrated high specificity of the Eurimmun ZIKV ELISA in European individuals with previous flavivirus exposure (either from infection or vaccination) including TBEV, DENV, and YFV [108]. However, a very recent study demonstrated high levels of cross-reactivity using commercially available DENV ELISA kits [109]. In this study, 100% of convalescent samples from NAAT confirmed ZIKV-infected individuals demonstrated cross-reactivity with DENV [109]. Although there is still some risk of cross-reactivity, the plaque reduction neutralization test remains the most specific serological method for diagnosing flaviviruses, including ZIKV [104]. An ELISA using the ZIKV NS1 antigen has been proposed as a more specific serological test [110]. In an initial study of sera from individuals with either RT-PCR confirmed or suspected ZIKV infection, sensitivity of combined IgG and IgM ELISA was 100% if samples were obtained ≥6 days post-symptom onset [110]. Furthermore, there was extremely limited cross-reactivity with sera from known DENV, WNV, JEV infected or YFV vaccinated individuals (specificity of 99.8%) [110]. The significant cross-reactivity demonstrated by most serological tests has hindered diagnosis in areas were multiple flaviviruses co-circulate. Development of serological tests with improved specificity is essential for assessing prevalence of ZIKV infection and aiding in diagnosis. 

### 7.2. NAAT

NAAT to detect ZIKV RNA in the serum, whole blood, urine, and cerebrospinal fluid (CSF) are available [105]. While NAAT are more specific than serological tests, they are limited by the short duration of RNAemia and RNAuria. In general, ZIKV RNA is detectable from serum for approximately seven days post-infection and in urine for approximately 15–20 days post-infection [111]. The sensitivity of different NAAT varies. In a comparison of seven published and two new RT-PCR assays, Corman et al. found that some published RT-PCR assays may be of limited value for diagnostics in the current outbreak due to lack of sensitivity or difficulty in obtaining necessary reagents [112]. Currently, 12 molecular assays are available through EUA [106].

## 8. Vaccines

It is widely accepted that vaccines provide a cost-effective method of preventing infectious diseases [113]. Given the rapid spread of and severe outcomes associated with ZIKV infection, the development of a safe and efficacious vaccine is critical. The existence of successful vaccines against other flaviviral diseases (YFV, JEV, DENV, and TBEV) indicates that it is possible to develop a vaccine against ZIKV. Forty-five ZIKV vaccine candidates consisting of multiple vaccine platforms are currently under consideration and at various stages of development as summarized in the WHO vaccine pipeline tracker [114]. Five candidate vaccines, including inactivated whole organism, DNA, synthetic peptide, and mRNA platforms are already in Phase I clinical trials (Table 1) and larger Phase II and III studies are planned pending the results of the Phase I trials [21]. Due to the large number of vaccine candidates under investigation, this review will focus on those in clinical trials or for which animal model data is available.

### 8.1. Inactivated Whole Organism (with or without Adjuvant)

Several developers are focusing on inactivated whole organism vaccine candidates [114]. Inactivated vaccines against flaviviruses including JEV and TBEV have been used successfully providing support for this method [115,116]. Benefits of an inactivated whole organism vaccine include multiple antigenic targets and non-replicating virus, which may improve safety. Purified inactivated virus (PIV) derived from a Puerto Rican strain of ZIKV was tested in Balb/c mice which received 1 µg of PIV vaccine with alum by either the intramuscular or subcutaneous route or sham inoculated with alum alone [117]. The PIV vaccine was shown to induce ZIKV-specific neutralizing antibodies after a single immunization and complete protection against ZIKV viremia was observed in those mice that received intramuscular injection [117]. It is important, however, as mentioned above, to note the limitations of this mouse model in recapitulating human disease. In recent studies by Abbink et al., 16 rhesus monkeys were immunized subcutaneously with 5 µg of ZIKV PIV with alum as an adjuvant or sham inoculated. It was demonstrated that all PIV inoculated animals developed both E protein-specific antibodies as well as neutralizing antibodies [79]. Furthermore, all PIV-inoculated monkeys were protected against challenge with wild-type ZIKV and had no detectable virus by RT-PCR in blood, urine, CSF, colorectal, or cervicovaginal secretions [79]. All sham inoculated monkeys had detectable viremia and in the majority, virus was detected in other body fluids as well [79]. In order to improve vaccine accessibility and reduce manufacturing costs, Yang et al. very recently proposed a cDNA clone-launched platform for high yield production of inactivated ZIKV vaccines [118]. Currently, Phase I trials of anti-ZIKV PIV vaccine candidates are underway (Table 1) [114,119]. 

### 8.2. DNA

DNA vaccines have been in development since the early 1990s and consist of a selected gene sequence cloned into a plasmid backbone. The plasmid is injected allowing DNA to be taken up by antigen presenting cells, which then express the plasmid-encoded genes to generate the target antigen(s) [120]. A candidate DNA vaccine against WNV was previously tested in humans and demonstrated excellent safety and immunogenicity [121,122]. A monovalent (DENV1) DNA vaccine candidate was also found to have an excellent safety profile in a Phase I clinical trial; however, it did not induce high levels of neutralizing antibodies [123]. Multiple ZIKV DNA vaccine candidates are in Phase I clinical trials (Table 1) [114,124]. These DNA vaccine candidates encode the *prM/E* genes of ZIKV. The E protein, in particular, is thought to be a major antigen against which neutralizing antibodies are produced and is considered important for protective efficacy in flaviviral vaccines [21]. In mice, a single immunization with DNA vaccine candidate encoding prM/E elicited higher E-specific antibody titers than did a DNA vaccine candidate encoding only E indicating the importance of prM in immunogenicity [117]. Furthermore, the prM/E DNA vaccine induced CD4+ and CD8+ T lymphocyte responses against E as demonstrated by increased IFN-γ production by intracellular cytokine staining [117]. Multiple anti-ZIKV DNA vaccine candidates induce both binding and neutralizing anti-ZIKV antibodies in rhesus monkeys [79,80,125]. 

### 8.3. RNA

RNA vaccines contain an open reading frame encoding the antigen of interest which is then translated by the host cellular machinery [126]. Because there is not the potential for genome integration, RNA-based vaccines may have a safety advantage over DNA vaccines [102]. Immunogenicity of several RNA vaccine candidates in animal models have been reported in the literature [102,103,127]. A lipid nanoparticle encapsulated modified mRNA encoding prM/E from an Asian lineage ZIKV strain was shown to protect both AG129 mice as well as C57BL/6 mice treated with blocking anti-IFNAR1 antibody (to create a lethal challenge model) [127]. Richner et al., further modified the mRNA to delete an immunodominant epitope within the E domain II fusion loop and demonstrated that the fusion loop mutant elicited serum antibody responses and protected against ZIKV challenge in mice. Furthermore, the antibodies elicited by the fusion loop mutant caused less ADE of DENV1 infection in cell culture and less immune enhancement of DENV2 infection in AG129 mice [127]. Another recently reported anti-ZIKV RNA nanoparticle vaccine candidate encoding the prM/E proteins of an Asian lineage ZIKV induced both antibody and CD8+ T cell responses in C57BL/6 mice [102]. Furthermore, in the rhesus macaque model a single immunization of a different ZIKV prM/E encoding mRNA lipid nanoparticle vaccine demonstrated neutralizing antibody titers that were fifty times greater than those induced by a single immunization of a DNA vaccine and more than twice as high as those induced by two immunizations of a DNA vaccine measured using the same assay in the same laboratory [103,125]. These results indicate promise for RNA anti-ZIKV vaccine candidates. There is currently a Phase I clinical trial of an mRNA vaccine candidate underway (Table 1) [114].

### 8.4. Recombinant Viral Vector

In rhesus monkeys, a rhesus adenovirus serotype 52 (RhAd52) vector-based vaccine elicited ZIKV-specific neutralizing antibodies following a single immunization which demonstrated a substantial breadth of antibody responses against linear ZIKV E protein epitopes (peptide microarray assays) [79]. Furthermore, this vaccine candidate protected against challenge with wt ZIKV as demonstrated by lack of detectable viral RNA in plasma [79]. Very recently, a recombinant vesicular stomatitis virus (rVSV) anti-ZIKV vaccine was tested in mice and demonstrated maternal protective immunity in challenged newborn mice born to vaccinated mothers [101]. Betancourt et al. investigated multiple rVSV expressing ZIKV E or prM/E constructs in mice and identified an attenuated VSV with mutated matrix protein expressing prM/E (VSVm-ZprME) that induced high neutralizing anti-ZIKV antibody titers as well as IFN-γ production by CD8+ T cells [101]. Furthermore, in a neonatal mouse challenge model, seven-day-old mice born to VSVm-ZprME vaccinated mothers were partially protected against neurological manifestations of ZIKV infection following challenge [101]. Additional recombinant viral-vectored anti-ZIKV vaccine candidates are in the pre-clinical stages of development [114]. 

Additional vaccine candidate platforms in pre-clinical studies include live-attenuated vaccines, recombinant subunit vaccines, peptide vaccines, and ZIKV exosome vaccines [114].

## 9. Therapeutics

There are currently no drugs approved for the treatment of ZIKV-infection. The aim of drug development is primarily to reduce viral load, reduce symptoms, and protect the unborn fetus from neurological sequelae [128]. Multiple studies have focused on “re-purposing” existing compounds for the treatment of ZIKV [129,130,131,132,133,134]. Zmurko et al. tested multiple compounds, including ribavirin and polymerase inhibitor 7-deaza-2′-C-methyladenosine (7DMA), to identify potential anti-ZIKV therapeutics [134]. Of the compounds tested, 7DMA inhibited ZIKV replication in vitro, and, when administered for 10 consecutive days (beginning 1 h prior to infection), reduced viremia as well as delayed time to disease progression in ZIKV-infected AG129 mice [134]. While 7DMA was well tolerated by the mice, there was only modest reduction in viremia and the initiation of treatment prior to infection is impractical in non-research settings. Rapid, high-throughput screening of drug/compound libraries has also been utilized in an attempt to identify compounds with in vitro anti-ZIKV activity [129,131]. In a large screen of 727 compounds using a high-throughput cell-based assay to screen for anti-ZIKV activity, ZIKV was found to be sensitive to pyrimidine synthesis inhibitors (e.g., brequinar) [129]. Furthermore, Barrows et al. screened 774 FDA-approved drugs for anti-ZIKV activity and identified over 20 compounds, including mycophenolate mofetil, daptomycin, and sertraline, that reduced viral infection in vitro [131]. In an even larger screen, ~6000 compounds, including approved drugs, clinical trial drug candidates, and pharmacologically active compounds, were tested to determine their ability to either inhibit ZIKV infection or suppress infection-induced caspase-3 activity in neuronal cells [133]. Of the compounds screened in this study, emricasan, a pan-caspase inhibitor, was the most potent anti-cell-death compound and it demonstrated neuroprotective activity for human neuronal progenitor cells, but did not suppress ZIKV replication [133]. Additionally, several anti-malarial compounds were identified as having anti-DENV2 and anti-ZIKV properties using a cell-based cytotoxicity assay [130]. Bullard-Feibelman et al. demonstrated that an FDA-approved hepatitis C virus (HCV) anti-viral, sofosbuvir, inhibited ZIKV replication and infection in tissue culture as well as protected mice from ZIKV-induced death [132]. However, it is important to note that in this study mice were treated one day after inoculation with ZIKV, a time-line that is not practical in human infections. 

Although anti-ZIKV therapeutic agents would likely be a welcome addition to the armamentarium, there are multiple challenges that face their development. Because clinical infection with ZIKV is typically mild, the primary populations for whom treatment would be indicated are pregnant women and those at increased risk for neurological complications, such as GBS. There are multiple ethical considerations in the development of therapeutics to be used during pregnancy. In general, pregnant women are excluded from clinical trials of new investigational compounds. In this setting, the inclusion of pregnant women would be warranted; however, the agent would need to be low risk to the mother, low risk to the fetus (not teratogenic), effective in preventing adverse fetal outcomes, and practical for use in resource-limited settings [128]. 

## 10. Conclusions

ZIKV is a rapidly emerging virus with a complex clinical picture. To date, much progress has been made in understanding the epidemiology and pathogenesis as well as developing vaccines which are essential to addressing the spread of ZIKV. However, many questions remain and a sustained global effort is required to battle this public health threat. Among the major issues requiring attention are the need for: (a) an improved understanding of the epidemiology including the potential interaction of ZIKV infection with other flaviviral infections; (b) an improved understanding of the clinical risk factors and mechanisms of severe disease; (c) identification of immunological correlates of protection which may aid in the rapid development of vaccines; and (d) the development of additional animal models which faithfully recapitulate human disease to better understand ZIKV pathogenesis and facilitate the development of countermeasures. In sum, an improved understanding of the epidemiology, pathogenesis, clinical risk factors for severe manifestations, and immunological correlates of protection are critical in developing effective countermeasures against this pathogen, including vaccines and therapeutics.

## Figures and Tables

**Table 1:** Summary of anti-Zika virus (ZIKV) vaccine candidates currently in clinical trials.

Type of Vaccine	Developers/Collaborators	Candidate Vaccine Name (If Available)	Stage of Development	Clinical Trial Registration Number
Inactivated whole organism	WRAIR/BIDMC/Harvard/NIAID/Sanofi Pasteur		Clinical (Phase 1)	NCT02963909
				NCT02952833
				NCT02937233
DNA	GeneOne Life Science, Inc/Inovio Pharmaceuticals	GLS-5700	Clinical (Phase I)	NCT02809443
				NCT02887482
DNA	VRC/NIAID	VRC ZIKV DNA	Clinical (Phase I)	NCT02840487
				NCT02996461
Synthetic peptide	NIAID	AGS-v	Clinical (Phase I)	NCT03055000
Measles-vectored	Themis Bioscience	MV-ZIKA	Clinical (Phase I)	NCT02996890
mRNA	Valera (Moderna)	mRNA-1325	Clinical (Phase I)	NCT03014089

WRAIR: Walter Reed Army Institute of Research, BIDMC: Beth Israel Deaconess Medical Center, NIAID: National Institutes of Allergy and Infectious Diseases, VRC: Vaccine Research Center.
